# An integrated workflow for *Mycoplasma* detection and removal from cell cultures and viral stocks

**DOI:** 10.1002/cpz1.70496

**Published:** 2026-10-30

**Authors:** Eliza Flores‐Souza, Carolina Colombelli Pacca‐Mazaro, Naiara Clemente Tavares, Camila Salles Nascimento, Tamiris Vanessa Miguel de Souza, Anna Beatriz de Oliveira Mendes, Erna Geessien Kroon, Carlos Eduardo Calzavara‐Silva, Jaquelline Germano de Oliveira

**Affiliations:** ^1^ Grupo de Pesquisa em Imunologia Celular e Molecular Instituto René Rachou‐Fiocruz Minas 30190‐002, Belo Horizonte Minas Gerais Brazil; ^2^ Instituto Nacional de Ciência e Tecnologia em Poxvírus Brazil; ^3^ Rede Mineira de Antivirais MG Brazil; ^4^ Laboratório de Vírus, Departamento de Microbiologia, Instituto de Ciências Biológicas Universidade Federal de Minas Gerais Av. Antônio Carlos, 6627, Belo Horizonte 31270‐901 Belo Horizonte MG Brazil; ^5^ These authors contributed equally to this work

**Keywords:** cell culture, Mycoplasma, Mycoplasma decontamination, qPCR, virus stock

## Abstract

Contamination of cell cultures and viral stocks with *Mycoplasma* is one of the most frequent and underdiagnosed problems in laboratories working with these materials, severely affecting experimental reproducibility, data reliability, and biosafety. This article presents an integrated set of robust, sensitive, and broad applicable protocols for the detection, treatment, and mitigation of *Mycoplasma* contamination in cellular and viral systems. Basic Protocol 1 describes a sensitive qPCR‐based method for *Mycoplasma* detection using DNA extracted from cell cultures and viral suspensions, including clear interpretation criteria based on amplification and melting curve analyses. Basic Protocol 2 outlines a sequential antibiotic treatment strategy for eliminating *Mycoplasma* from contaminated cell cultures, combining different antimicrobial classes and continuous monitoring of cell viability and morphology. Basic Protocol 3 presents a complementary approach to reduce *Mycoplasma* burden in viral stocks through serial dilution and controlled replication, aiming to preserve viral infectivity while minimizing bacterial contaminants. Together, these protocols provide a practical and reproducible workflow that enables rapid identification of contamination, implementation of effective corrective measures, and molecular validation of decontamination. The procedures are suitable for virology, cell biology, and experimental virus production laboratories, thereby improving experimental quality and data reliability. © 2026 The Author(s). *Current Protocols* published by Wiley Periodicals LLC.

**Basic Protocol 1**: Sensitive detection of *Mycoplasma* contamination in cell cultures and viral stocks by qPCR

**Basic Protocol 2**: Treatment of cell cultures for *Mycoplasma* decontamination

**Basic Protocol 3**: Elimination of *Mycoplasma* contamination in viral stocks using serial dilution

## INTRODUCTION

Cell cultures are widely used as experimental models to study physiological and pathological processes, including toxicity assessment, drug discovery and development, vaccine production, and virus replication for diagnostic and research purposes (Segeritz & Vallier, 2017).

The reliability of results from these systems depends on strict quality control and maintaining the biological integrity of cell cultures. The presence of microbiological contaminants, such as fungi, bacteria, and particularly *Mycoplasma* contamination, can significantly impair cell culture quality by affecting essential cellular functions, including metabolism, proliferation rate, viability, gene expression, and cellular responses to experimental stimuli (Huang *et al.*, 2023). These changes can increase experimental variability, complicate data interpretation, and reduce reproducibility, ultimately weakening the reliability and validity of the results. Members of the genus *Mycoplasma* are bacteria in the class Mollicutes, characterized by the absence of a cell wall, small size, and compact genomes, resulting in limited metabolic capacity and a high dependence on nutrients supplied by host cells (Nicolson, 2019). Due to their physical properties, particularly their small size and high morphological plasticity, these microorganisms may not be retained by 220‐nm pore filters commonly used in cell culture systems. In addition, the lack of a rigid cell wall renders antibiotics targeting bacterial cell wall synthesis ineffective in controlling this type of contamination (Young et al., 2010).

These features allow *Mycoplasmas* to adapt well to in vitro systems and persist in cell cultures without obvious changes in the medium or cell morphology, making them difficult to detect by routine methods (Jean et al., 2017). Despite the absence of visible signs, *Mycoplasma* contamination can induce profound alterations in cellular physiology, including changes in energy metabolism, nucleic acid and protein synthesis, gene expression regulation, and cell proliferation rates, as well as direct interference with transfection efficiency, drug responses, and viral replication (Huang et al., 2023; Leonovich, 2024).

In cultures used for viral replication, these effects can reduce infectivity, affect genetic stability, and impair assay reproducibility, impacting both experimental research and biotechnological applications. Therefore, *Mycoplasma* contamination poses a serious risk to the integrity of cell and viral stocks, reinforcing the need for sensitive detection strategies and effective decontamination methods (Malenovska & Reichelova, 2011).

*Mycoplasma* detection in cell cultures can be carried out using different approaches, mainly divided into nucleic acid–based methods, such as polymerase chain reaction (PCR), and biochemical assays that evaluate metabolic activity or cell viability. Luciferase‐based biochemical assays detect the activity of *Mycoplasma*‐specific metabolic enzymes by producing a luminescent signal and are considered rapid, simple, and easy to implement. A key advantage is that they indicate the presence of viable mycoplasma*s*, since the signal depends on active metabolism. Therefore, these assays are especially useful when viability is critical, such as in evaluations after antibiotic treatment (Falagan‐Lotsch et al., 2015).

However, biochemical assays also present relevant limitations. Their reliance on enzymatic activity may yield false‐negative results if the target enzymes are present at low abundance, are metabolically inactive, or vary across *Mycoplasma* species (Falagan‐Lotsch et al., 2015). In addition, these methods may be less sensitive than qPCR under certain conditions. Although qPCR can detect residual DNA from nonviable mycoplasma*s*, leading to positive results even after successful treatments, it provides greater robustness, standardization, and flexibility for quantitative analyses and longitudinal monitoring when used with proper controls and standard curves (Falagan‐Lotsch et al., 2015; Uphoff et al., 2012; Young et al., 2010). Thus, a combined approach, using qPCR as the primary screening method and biochemical assays to assess viability, can improve the reliability of the results.

Quantitative polymerase chain reaction (qPCR) is widely used as a reference method for *Mycoplasma* detection due to its high sensitivity, specificity, and rapid turnaround, enabling direct identification of *Mycoplasma* DNA without the need for prolonged culture (Falagan‐Lotsch et al., 2015; Uphoff et al., 2012). It can detect very low DNA levels and shows strong agreement with classical microbiological methods, often outperforming colorimetric and culture‐based assays (Young et al., 2010). qPCR protocols targeting conserved regions, such as the 16S rRNA gene, allow detection of most *Mycoplasma* species associated with cell culture contamination, making this approach well suited for routine laboratory monitoring (Kong et al., 2001). The primers used in this study MGSO (5′‐TGC ACC ATC TGT CAC TCT GTT AAC CTC‐3′) and GPO‐3 (5′‐GGG AGC AAA CAG GAT TAG ATA CCC T‐3′) target conserved regions of the 16S rRNA gene and exhibit broad specificity for *Mycoplasma* species commonly associated with cell culture contamination, including at least 14 formally described species. This primer pair generates a 288–base pair amplicon, as previously reported (Kong et al., 2000, 2001). Sequence analysis revealed similarity to multiple members of the genus *Mycoplasma*, including species such as *M. seminis*, *M. anserisalpingitidis*, *M. aquilae*, *M. tauri*, *M. enhydrae*, *M. iguanae*, *M. spermatophilum*, and *Candidatus Mycoplasma mahonii*, as well as unclassified *Mycoplasma* sp., indicating broad assay reactivity across the genus.

These protocols aimed to detect *Mycoplasma* in cell cultures and viral stocks by qPCR using universal primers targeting a conserved region of the 16S rRNA gene, and to establish a decontamination protocol based on limiting dilution combined with treatment with ciprofloxacin hydrochloride and tylosin.

### Cautions

All plastic materials used must be new and sterile to prevent prior contamination with *Mycoplasma* genetic material.

All liquid reagents, including culture media, fetal bovine serum, antibiotics, and trypsin, must be previously tested for *Mycoplasma* by qPCR and yield negative results.

All procedures must be performed in an appropriate biosafety cabinet with efficient ventilation, and the operator must wear personal protective equipment.

If the virus stock undergoing decontamination is classified as a Biosafety Level 2 or 3 pathogen, all applicable guidelines and regulations for handling and using pathogenic microorganisms must be strictly followed.

The genetic material extraction kit must be used with caution to minimize the risk of cross‐contamination between samples and kit reagents.

All pipette tips used in this protocol must be filtered.

All waste materials potentially contaminated with *Mycoplasma* (culture media, pipette tips, tubes) must be decontaminated by autoclaving at 121°C for 30 min at 15 psi before disposal, or treated for at least 30 min with a freshly prepared 10% (v/v) dilution of commercial household bleach, corresponding to approximately 0.5% sodium hypochlorite.

*NOTE*: All protocols involving animals must be reviewed and approved by the appropriate Animal Care and Use Committee and must follow regulations for the care and use of laboratory animals. Appropriate informed consent is necessary for obtaining and use of human study material.

## Basic Protocol 1 SENSITIVE DETECTION OF *Mycoplasma* CONTAMINATION IN CELL CULTURES AND VIRAL STOCKS BY qPCR

In this protocol, the user will perform total DNA extraction from cell cultures and viral suspensions using a commercial kit for genetic material extraction, following standardized procedures for cell lysis, DNA purification, and elution. The method relies on efficient removal of proteins, lipids, and other contaminants, yielding purified DNA suitable for subsequent molecular analyses. At the end of the procedure, the user is expected to obtain intact DNA that is free of inhibitors and at a measurable concentration, which will be quantified by spectrophotometry (e.g., a NanoDrop instrument), enabling its use in qPCR assays for *Mycoplasma* detection.

### Materials

Cell samples (e.g., adherent mammalian cell line)
Viral stocks (assigned appropriate biosafety level)
PBS (phosphate‐buffered saline)
Trypsin–EDTA (Gibco, cat. no. 25200‐072, Lot 2660235)
Culture medium (e.g., Dulbecco's Modified Eagle Medium (DMEM) (Sigma‐Aldrich, cat. no. D6046, Lot 31600034))
DNA extraction kit (e.g., PureLink Genomic DNA Mini Kit (Invitrogen; cat. no. K182002; Batch no. 2232335) – for human/microbial DNA extraction; Includes: Proteinase K, RNase A, Lysis/Binding Buffer, Wash Buffers 1 and 2, Elution Buffer, Spin Columns
96%–100% ethanol (molecular biology grade)
DNA‐free ultrapure water
PowerUp SYBR Green Master Mix (Applied Biosystems; cat. no. A25741)
Universal *Mycoplasma* qPCR primers (targeting ∼288 bp region of 16S rRNA genes): forward primer MGSO (10 µM): 5′ TGC ACC ATC TGT CAC TCT GTT AAC CTC‐3′; ′ reverse primer GPO ‐3 (10 µM): 5′‐GGG AGC AAA CAG GAT TAG ATA CCC T‐3′;
qPCR controls:
Positive *Mycoplasma* control DNA
No‐template control (NTC): nuclease‐free water (DNASE RNASE, Invitrogen cat. no. 10977015. Lot: 2186750.)

Biosafety cabinet (Class II biological safety cabinet)
Water bath or dry block heater set at 55°C
15‐ and 50‐ml Conical centrifuge tubes (Falcon)
1.5‐ml Microcentrifuge tubes, sterile
Microcentrifuge (e.g., Eppendorf) compatible with 1.5 ml tubes
Centrifuge for conical tubes (rotor for 15/50 ml tubes)
Ice and ice bucket
Filtered pipette tips (aerosol‐resistant)—various sizes
Adjustable pipettes (P10, P200, P1000)
spectrophotometer (e.g., NanoDrop ‐ Thermo Scientific; NanoDrop software (for DNA quantification readout)
Real‐time PCR instrument (e.g. QuantStudio 12K Flex or ViiA 7)
qPCR plates or tubes compatible with the PCR instrument
Plate seal or optical adhesive film
Computer with real‐time PCR analysis software

#### DNA extraction and qPCR‐based detection of Mycoplasma

##### Genomic DNA extraction

1 Preheat a water bath or heating block to 55°C.
2 For adherent mammalian cells (≤5 × 10⁶ cells), remove the culture medium, trypsinize the cells, and resuspend them in complete culture medium.
3 Transfer the cell suspension to a conical centrifuge tube and centrifuge at 250 × *g* for 5 min to pellet the cells.
4 Discard the supernatant and resuspend the cell pellet in 200 µl of 1× PBS.
5 For viral samples, thaw a viral aliquot on ice.
  Thawing on ice helps preserve nucleic acid integrity.
6 Centrifuge the viral suspension for 5 min at 250 × *g* and room temperature (RT).
7 Transfer 50 µl supernatant to a microcentrifuge tube containing 150 µl of 1× PBS.
8 Add 20 µl Proteinase K to each tube containing mammalian cells or viral suspensions.
9 Add 20 µl RNase A to each tube.
  RNaseA removes residual RNA, improving DNA purity.
10 Incubate the samples at room temperature for 2 min.
11 Add 200 µl PureLink Genomic Lysis/Binding Buffer to each tube and mix gently by pipetting.
12 Incubate at 55°C for 10 min to promote cell lysis and protein digestion.
13 Add 200 µl of 96%–100% ethanol to each lysate and mix carefully by inversion.

###### DNA binding

14 Place a PureLink Spin Column into a collection tube provided with the kit.
15 Transfer the entire lysate (∼640 µl) to the Spin Column.
16 Centrifuge1 min at 10,000 × *g* and RT.
17 Discard the collection tube and place the column into a new, clean collection tube.

###### DNA washing

18 Add 500 µl Wash Buffer 1 to the column.
19 Centrifuge 1 min at 10,000 × *g* and RT.
20 Discard the collection tube, then transfer the column to a new one.
21 Add 500 µl Wash Buffer 2 to the column.
22 Centrifuge 3 min at maximum speed and RT.
23 Discard the collection tube.
  Complete removal of residual ethanol is essential to prevent inhibition of downstream applications.

###### DNA elution

24 Transfer spin column to a sterile 1.5‐ml microcentrifuge tube.
25 Add 100 µl PureLink Genomic Elution Buffer directly to the center of the column membrane.
26 Incubate for 1 min at RT and centrifuge 1 min at maximum speed and RT.
27 To recover additional DNA, perform a second elution by adding the same volume of elution buffer to a new sterile microcentrifuge tube.
28 Centrifuge 1.5 min at maximum speed and RT, then discard the column.

###### DNA quantification using NanoDrop

29 Turn on the NanoDrop spectrophotometer and the connected computer.
30 Open the NanoDrop software and select the DNA quantification option.
31 Clean the pedestal and optical arm according to the manufacturer's instructions.
32 Add 1 µl ultrapure water to the pedestal and perform a blank measurement.
33 Clean the pedestal and add 1 µl DNA sample.
34 Click “Measure” and record DNA concentration and A260/A280 ratio.
35 Repeat the measurement for all samples.
  Pure DNA samples typically display an A260/A280 ratio ≥1.8.

###### qPCR‐based Mycoplasma detection

36 In a PCR‐dedicated clean hood, prepare a master mix for each reaction containing:
  5 µl PowerUp SYBR Green Master Mix (2×)
  1 µl MGSO forward primer (10 µM)
  1 µl GPO‐3 reverse primer (10 µM)
  1 µl DNase/RNase‐free water per reaction.
  Prepare sufficient volume for all reactions to be performed.
37 Purified DNA (2 µl; corresponding to approximately 5–20 ng total DNA) is added separately to each well in a subsequent step.
38 Gently mix by slow pipetting and briefly centrifuge for 15 sec to remove air bubbles.
39 Dispense 8 µl master mix into each well of a qPCR plate.
40 In a separate area designated for DNA handling, add 2 µl purified DNA to the bottom of each corresponding well.
  Avoid releasing the pipette plunger before removing the tip to prevent bubble formation.
41 Include a *Mycoplasma*‐positive control (Sartorius; Cat. No. SMB95‐1005; Lot No. 9515P22A1) and a no‐template control containing nuclease‐free water.
42 Seal the plate with an optical adhesive film and cover with aluminum foil.
43 Centrifuge the plate for 20 sec to ensure proper liquid positioning at the bottom of the wells.
44 Place the plate into the real‐time PCR instrument and set the following cycling conditions:

**Initial denaturation**:

95°C for 10 min

**Amplification (40 cycles)**:

95°C for 15 sec
60°C for 30 sec
72°C for 10 sec

**Melting curve analysis**:

95°C for 10 sec
65°C for 60 sec
95°C for 1 sec

45 Start the qPCR run and analyze amplification and melting curves at the end of the reaction.
  The expected outcome is the specific detection of Mycoplasma DNA in contaminated samples, indicated by a sigmoidal amplification curve and a characteristic melting profile. Purified DNA may be used for additional analyses, and positive samples should be directed to appropriate decontamination or disposal protocols.

## Basic Protocol 2 TREATMENT OF CELL CULTURES FOR MYCOPLASMA DECONTAMINATION

This protocol describes a two‐cycle antibiotic‐based approach to eliminate *Mycoplasma* contamination in cell cultures. The method involves sequential cell passaging in media supplemented with tylosin followed by ciprofloxacin, aiming to remove *Mycoplasma* while preserving cell viability, morphology, and growth. When necessary, the complete treatment sequence may be repeated depending on the initial level of *Mycoplasma* contamination and the results of qPCR monitoring. Successful treatment results in *Mycoplasma*‐free cell cultures, with decontamination confirmed by qPCR as described in Basic Protocol 1.

### Materials

Cell samples (example: BSC‐40 cells)
Culture medium (e.g., Dulbecco's Modified Eagle Medium (DMEM)) (Sigma‐Aldrich, cat. no. D6046, Lot 31600034)
Antibiotic–Antimycotic (Anti‐Anti; Penicillin (100 U/ml), streptomycin (100 μg/ml), and amphotericin B (0.25 μg/ml)) (Gibco, cat. no.15240‐062, Lot 1712153)
Fetal bovine serum (FBS), 5% (Cultilab, cat. no. F0630)
Phosphate‐buffered saline (PBS)
Trypsin‐EDTA (Gibco, cat. no. 25200‐072)
Tylosin (8 µg/ml; SIGMA, cat. no. T‐3397)
Ciprofloxacin (10 µg/ml; Cristália, ANVISA registration number: 1.0298.0489)

T25 cell culture flasks (new and sterile)
Sterile serological pipettes (1, 5, and 10 ml)
Inverted microscope
Cell culture incubator (37°C, 5% CO₂)
Biological safety cabinet
Aluminum foil

#### Treatment of cell cultures for mycoplasma decontamination

Confirm *mycoplasma* positivity by qPCR using the same protocol and interpretation criteria described in Basic Protocol 1, including appropriate positive and negative controls.

Only cultures exhibiting specific amplification and a melting curve profile consistent with mycoplasma are considered mycoplasma‐positive; therefore, only these cultures should be subjected to the decontamination treatment.

Clearly label contaminated culture flasks and keep them physically separated from *mycoplasma*‐negative cultures throughout the entire procedure.

1 Preferentially select cell cultures at early passages for antibiotic treatment. After thawing, maintain the cells in culture until stable growth and normal cellular morphology are achieved before initiating the protocol, as the thawing process may transiently alter the cell cycle and cellular physiological characteristics.
2 Use exclusively new, small‐volume cell culture flasks (25 cm²) throughout the treatment.
  Smaller flasks reduce antibiotic use and limit contamination.
3 Carefully remove the culture medium, considering the possible presence of mycoplasma in the supernatant. Discard the medium into a container containing sodium hypochlorite, preferably a narrow‐neck bottle.
  This precaution minimizes aerosol formation and contamination of the biological safety cabinet and consumables.

#### Cycle 1: Tylosin treatment

4 Wash cell monolayer twice with PBS prewarmed at 37°C.
5 Perform cell trypsinization according to the standard protocol for the cell line used.
6 Discard excess trypsin and resuspend the cells in 3 ml culture medium supplemented with 5% FBS and containing tylosin (8 µg/ml) and Anti‐Anti 1×.
7 Transfer 1 ml cell suspension to a new sterile T25 flask and complete the volume with 4 ml of DMEM supplemented with 5% FBS, containing tylosin (8 µg/ml) and Anti‐Anti 1×.
8 Incubate at 37°C in a humidified atmosphere containing 5% CO₂.
9 When the monolayer reaches 100% confluence, repeat steps 4 through 8.
10 Complete three consecutive passages of the cells in medium containing tylosin.
  Multiple passages of the cells increase the efficiency of mycoplasma elimination.

#### Cycle 2: Ciprofloxacin treatment

11 After completing three passages in tylosin‐containing medium, perform three additional passages using culture medium supplemented with 5% FBS, containing ciprofloxacin (10 µg/ml) and Anti‐Anti 1×, following the same procedure.
12 Protect ciprofloxacin‐treated cultures from light by covering the flasks with aluminum foil throughout the incubation period.
  Ciprofloxacin is photosensitive and may cause phototoxicity. Marked alterations may indicate treatment‐associated cytotoxicity.
13 Incubate the cells at 37°C in a humidified atmosphere with 5% CO₂.
14 Observe the cultures daily under an inverted microscope to monitor changes in cell morphology, viability, and growth rate.
  Marked alterations may indicate treatment‐associated cytotoxicity.

#### Post‐treatment

15 After completion of the entire treatment period (Cycle 1 + Cycle 2), maintain the cells in culture medium free of tylosin and ciprofloxacin for at least one additional passage.
16 Extract DNA from the treated cultures and perform Mycoplasma detection by qPCR as described in Basic Protocol 1.
17 Cultures yielding a negative qPCR result should be maintained for subsequent passage(s) in medium free of the antibiotics used for decontamination (tylosin and ciprofloxacin) and retested by qPCR to confirm the sustained absence of Mycoplasma amplification
18 Consider the cell culture successfully decontaminated only when the absence of specific amplification is confirmed after post‐treatment passage. Persistently or recurrently positive cultures should undergo an additional treatment cycle or be discarded in accordance with biosafety regulations.
  Persistently positive cultures should be discarded **in accordance with** biosafety regulations.

## Basic Protocol 3 ELIMINATION OF *MYCOPLASMA* CONTAMINATION IN VIRAL STOCKS USING SERIAL DILUTION

This protocol describes the reduction and elimination of *Mycoplasma* contamination from viral stocks by propagating viruses in *Mycoplasma*‐free cell cultures using serial dilutions of the viral suspension combined with antibiotic treatment. The contaminated viral suspension is clarified, filtered, serially diluted from 10⁻¹ to 10⁻⁶ in culture medium containing ciprofloxacin and tylosin, and used to infect Mycoplasma‐free cell monolayers. Following viral adsorption, the cells are maintained in antibiotic‐containing medium and monitored for cytopathic effect. During the serial dilution process, *Mycoplasma* contamination in the samples decreases progressively, while the virus is propagated under controlled cell culture conditions. Viral material recovered from each dilution is subsequently tested for *Mycoplasma* by qPCR. The highest viral dilution that retains detectable viral infectivity while testing negative for *Mycoplasma* is selected for expansion. The number of serial dilutions and propagation cycles required for effective decontamination may vary depending on the initial level of *Mycoplasma* contamination and the results of qPCR monitoring. In our experimental experience, the cowpox virus (CPXV) stock was successfully decontaminated after a single cycle, whereas the other viral stocks required at least three cycles. At the end of the procedure, the resulting viral stocks are expected to be free of *Mycoplasma* contamination, which must be confirmed by qPCR analysis as described in Basic Protocol 1. When properly executed, this protocol enables the recovery of *Mycoplasma*‐free viral stocks suitable for experimental use and long‐term storage.

### Materials

*Mycoplasma*‐contaminated viral stock
*Mycoplasma*‐free permissive cells adapted to *in vitro* culture
Culture medium (e.g., DMEM) (Sigma‐Aldrich, cat. no. D6046)
Antibiotic–Antimycotic (Anti‐Anti; Penicillin (100 U/ml), streptomycin (100 μg/ml), and amphotericin B (0.25 μg/ml)) (Gibco, cat. no.15240‐062)
5% FBS (Cultilab, cat. no. F0630)
Phosphate‐buffered saline (PBS)
Trypsin‐EDTA (Gibco, cat. no. 25200‐072)
Trypan blue solution (0.4%) (Gibco, cat. no.15250‐061)
Ciprofloxacin hydrochloride (10 µg/ml, Cristália, ANVISA registration number: 1.0298.0489, see recipe)
Tylosin (8 µg/ml; SIGMA, cat. no. T‐3397) (see recipe)
Sterile water or DNase/RNase‐free water
Freshly prepared 10% (v/v) dilution of commercial household bleach, corresponding to approximately 0.5% Sodium hypochlorite solution, for biological waste decontamination

Six‐well cell culture plates, new and sterile
Conical tubes (Falcon)
1.5‐ or 2.0‐ml microcentrifuge tubes
1, 5, and 10‐ml sterile serological pipettes (1, 5, and 10 ml)
Sterile filtered pipette tips
Adjustable micropipettes
Neubauer hemocytometer
Inverted microscope
CO₂ cell culture incubator (37°C, 5% CO₂)
Refrigerated centrifuge
Ice
Biological safety cabinet
Personal protective equipment (gloves, lab coat, mask)
−70°C freezer

#### Elimination of mycoplasma from viral stocks by serial dilution and cell culture propagation

1 Confirm the presence of *mycoplasma* in the viral stock by qPCR as described in Basic Protocol 1, including appropriate positive and negative controls.
  Only viral stocks showing specific amplification and a melting curve consistent with mycoplasma should be subjected to this protocol.
2 Prepare 6‐well culture plates containing *mycoplasma*‐free permissive cells. Determine the cell concentration using trypan blue and a Neubauer hemocytometer, and seed at a density of approximately 2.0 × 10⁵ to 3.0 × 10⁵ cells per well in 2 ml culture medium supplemented with 5% FBS.
3 Incubate the plates at 37°C with 5% CO₂ until cells reach 80–90% confluence, typically within 18–24 hr.
  Proper confluence ensures efficient viral infection and cell viability during propagation.
4 In sterile tubes, prepare serial tenfold dilutions (10⁻¹ to 10⁻⁶) of the *mycoplasma*‐contaminated viral stock in supplemented culture medium containing ciprofloxacin (10 µg/ml) and tylosin (8 µg/ml). Use filtered pipette tips and change tips between each dilution to prevent cross‐contamination.
  Serial dilution progressively reduces the mycoplasma load present in the viral suspension.
5 Carefully remove the culture medium from each well and inoculate the cells with 200 µl each prepared viral dilution, ensuring that each dilution is applied to a separate well.
6 Incubate the plates at 37°C with 5% CO₂ for 1 hr, gently rocking the plates every 15 min to facilitate viral adsorption.
  Avoid drying the cell monolayer during the adsorption period.
7 Following adsorption, carefully add 1.8 ml culture medium supplemented with 5% FBS, ciprofloxacin (10 µg/ml) and tylosin (8 µg/ml) to each well, resulting in a final volume of 2 ml per well.
8 Protect the ciprofloxacin‐containing cultures from light and incubate the plates at 37°C with 5% CO₂, monitoring the cultures daily using an inverted microscope.
9 Daily assess cell morphology and the development of cytopathic effects consistent with the expected viral infection, avoiding progression to excessive cytotoxicity.
  Higher dilutions generally show reduced cytotoxicity and greater reduction of mycoplasma load.
10 At the peak of viral replication or at the predefined experimental time point, carefully collect the supernatants from each well into sterile tubes labeled according to the corresponding dilution.
11 Clarify the supernatants by centrifugation 5 min at 300 × g and RT, if necessary, to remove cellular debris, and transfer the clarified supernatants to new sterile tubes.
12 Aliquot the viral supernatants and immediately store them at −70°C until *mycoplasma* testing.
  Rapid freezing preserves viral integrity and prevents nucleic acid degradation.
13 Extract DNA from aliquots of the viral supernatants and perform mycoplasma detection by qPCR as described in Basic Protocol 1.
14 Select viral stocks derived from the highest dilutions that test negative for Mycoplasma by qPCR.
15 Subject the selected qPCR‐negative viral stock to controlled amplification in Mycoplasma‐free permissive cells.
16 Following viral expansion, extract DNA from an aliquot of the resulting viral stock and repeat Mycoplasma qPCR testing as described in Basic Protocol 1.
17 Consider the viral stock successfully decontaminated only when the absence of specific amplification is maintained after viral expansion. Stocks showing recurrent amplification should undergo an additional serial‐dilution and propagation cycle.

## Reagents and Solutions

Use sterile, DNA‐free ultrapure (Milli‐Q) water for all preparations unless otherwise stated.

### Antibiotic–Antimycotic (Anti‐Anti, 1×)

Antibiotic–Antimycotic (100×; Gibco, Thermo Fisher Scientific). Contains:
  Penicillin (10,000 U/ml)
  Streptomycin (10,000 µg/ml)
  Amphotericin B (25 µg/ml)
Dilute 1:100 in culture medium to obtain a final 1× concentration
Mix under aseptic conditions
Store stock solution at −20°C, protected from light
After dilution in culture medium, store at 4°C for up to 30 days

### Ciprofloxacin (10 µg/ml working solution)

Ciprofloxacin hydrochloride powder (pharmaceutical‐grade supplier or Sigma‐Aldrich)
Prepare stock solution in sterile water or DNase/RNase‐free water according to manufacturer's instructions
Filter‐sterilize using a 0.22‐µm filter
Dilute in culture medium to a final concentration of 10 µg/ml
Store stock solution at −20°C, protected from light
Avoid light exposure during use, as the reagent is photosensitive

### DMEM supplemented with 5% fetal bovine serum and Antibiotic–Antimycotic

Dulbecco's Modified Eagle Medium (DMEM) (Sigma‐Aldrich, cat. no. D6046, Lot 31600034)
Fetal bovine serum (FBS), 5% (v/v; Gibco or equivalent)
Antibiotic–Antimycotic, 1× (Gibco; see above)
Prepare under aseptic conditions
Store at 4°C for up to 30 days

### PBS

PBS 10× solution (Thermo Fisher Scientific or equivalent)
Dilute in sterile distilled water to a final 1 × concentration
Sterilize by filtration (0.22 µm), if required
Store at room temperature or 4°C for up to 6 months

### Trypsin–EDTA

Trypsin–EDTA 0.25% (Gibco or equivalent)
Ready to use
Store up to 1 year at −20°C
After thawing, keep at 4°C and use within 30 days

### Tylosin (8 mg/ml working solution)

Tylosin powder (Sigma‐Aldrich, cat. no. T3397)
Prepare a stock solution in sterile water
Sterilize using a 0.22‐µm filter
Dilute in culture medium to a final concentration of 8 µg/ml
Store stock solution up to 1 year at −20°C

## Commentary

### Critical Parameters

The successful and reproducible application of protocols for *Mycoplasma* detection and treatment relies on several critical parameters that must be carefully controlled throughout the procedure. These parameters are primarily related to sample quality, cell culture conditions, reagent preparation and handling, and proper execution of experimental steps.

#### Sample quality and condition

Using cell cultures and viral stocks under optimal conditions is essential for protocol success. Cells should be used at early passages, exhibiting preserved morphology and stable growth before treatment initiation. Cultures severely compromised by *Mycoplasma* contamination or displaying overt signs of cellular stress may respond poorly to antibiotic treatment, thereby impairing complete elimination of the contaminant.

#### Cell culture conditions

Strict maintenance of culture conditions (37°C, 5% CO₂, and appropriate fetal bovine serum concentration) is critical throughout the treatment process. Abrupt changes in culture medium composition, serum concentration, or environmental conditions may negatively affect cell viability and interfere with treatment efficacy. The use of small‐volume culture flasks is recommended to reduce antibiotic consumption and improve treatment control.

#### Antibiotic preparation and use

Antibiotic concentrations used during the treatment cycles must be strictly adhered to. Suboptimal concentrations may lead to ineffective treatment, whereas excessive concentrations may induce cytotoxicity and promote the selection of resistant cell subpopulations. Antibiotics should be prepared using sterile solutions, stored according to manufacturer recommendations, and protected from light when applicable, as with ciprofloxacin.

#### Prevention of cross‐contamination

Handling contaminated cultures poses a significant risk of *Mycoplasma* dissemination within the laboratory. Contaminated flasks must be clearly labeled and physically separated from *Mycoplasma*‐negative cultures throughout the procedure. Culture media and waste should be disposed of using appropriate disinfectant solutions, such as hypochlorite, to minimize environmental contamination.

#### qPCR monitoring

Confirmation of the presence or absence of *Mycoplasma* by qPCR is a critical decision point in the protocol. Including appropriate positive and negative controls, along with careful analysis of amplification and melting curves, is essential to avoid false‐positive or false‐negative results. Because the analytical limit of detection (LoD) was not experimentally established for the qPCR conditions used in this protocol, a negative result does not completely rule out residual *Mycoplasma* DNA below the assay detection limit. Therefore, negative results obtained immediately after treatment should be confirmed after subsequent cell passage or viral stock expansion. Only samples showing sustained absence of specific amplification during post‐treatment monitoring should be considered successfully decontaminated.

#### Serial dilution and Mycoplasma load

When applicable, serial dilution of viral stocks reduces viral titer and, in turn, decreases *Mycoplasma* load in the samples. However, dilution alone should not be considered a definitive decontamination strategy and must always be complemented by molecular confirmation of the absence of contaminants.

### Troubleshooting Table

Table 1 summarizes the most common problems encountered during *Mycoplasma* detection and treatment in cell cultures and viral stocks, along with their possible causes and recommended solutions.

**Table 1:** Troubleshooting guide for mycoplasma detection and elimination in cell cultures and viral samples

Problem	Possible cause	Solution
Weak or absent qPCR amplification	Low DNA concentration or DNA degradation	Assess DNA quality and concentration; repeat DNA extraction if necessary
Nonspecific melting curve	Amplification of nonspecific products	Optimize annealing temperature and confirm primer specificity
Positive signal in no‐template control	Cross‐contamination during reaction setup	Prepare master mix in a PCR‐dedicated clean area and replace tips and reagents
Marked cytotoxicity during treatment	Excessive antibiotic concentration	Verify antibiotic concentrations and reduce dosage if required
Slow cell growth after treatment	Antibiotic‐induced cellular stress	Increase recovery time between passages and monitor cell viability
Persistent *Mycoplasma* contamination	Insufficient number of antibiotic passages	Repeat the treatment cycle as described in the protocol
Irreversible morphological alterations	Severely compromised culture	Discard the culture and replace with *Mycoplasma*‐free cells
Recontamination after treatment	Improper handling or contaminated environment	Strengthening biosafety practices and physically separate positive and negative cultures

### Understanding Results

Proper execution of the protocols described in this manuscript enables reliable detection of Mycoplasma contamination, evaluation of treatment efficacy, and confirmation that cell cultures and/or biological stocks are free of this contaminant. Interpretation of results should always consider appropriate controls, assay reproducibility, and combined evaluation of molecular and phenotypic parameters.

#### Expected results for Basic Protocol 1 (Mycoplasma detection by qPCR)

*Mycoplasma*‐contaminated samples exhibit specific amplification during qPCR, typically detected at early or intermediate cycles depending on the initial contaminant load. These samples display a single, reproducible melting curve consistent with the expected *Mycoplasma* amplicon, allowing clear distinction between specific amplification and nonspecific products.

Positive controls must consistently amplify within the expected *Ct* range and present a characteristic melting curve. No‐template controls should show no amplification or only late, inconsistent signals without a defined melting peak. Failure to meet these criteria invalidates the run and requires repetition of the assay.

Reliable negative results are characterized by the complete absence of specific amplification across multiple technical and biological replicates (Figure 1). Ambiguous results, such as late amplification with atypical melting profiles, may indicate residual contamination, a low *Mycoplasma* burden, or nonspecific amplification and should be confirmed by repeating DNA extraction and qPCR.

**Figure 1:**
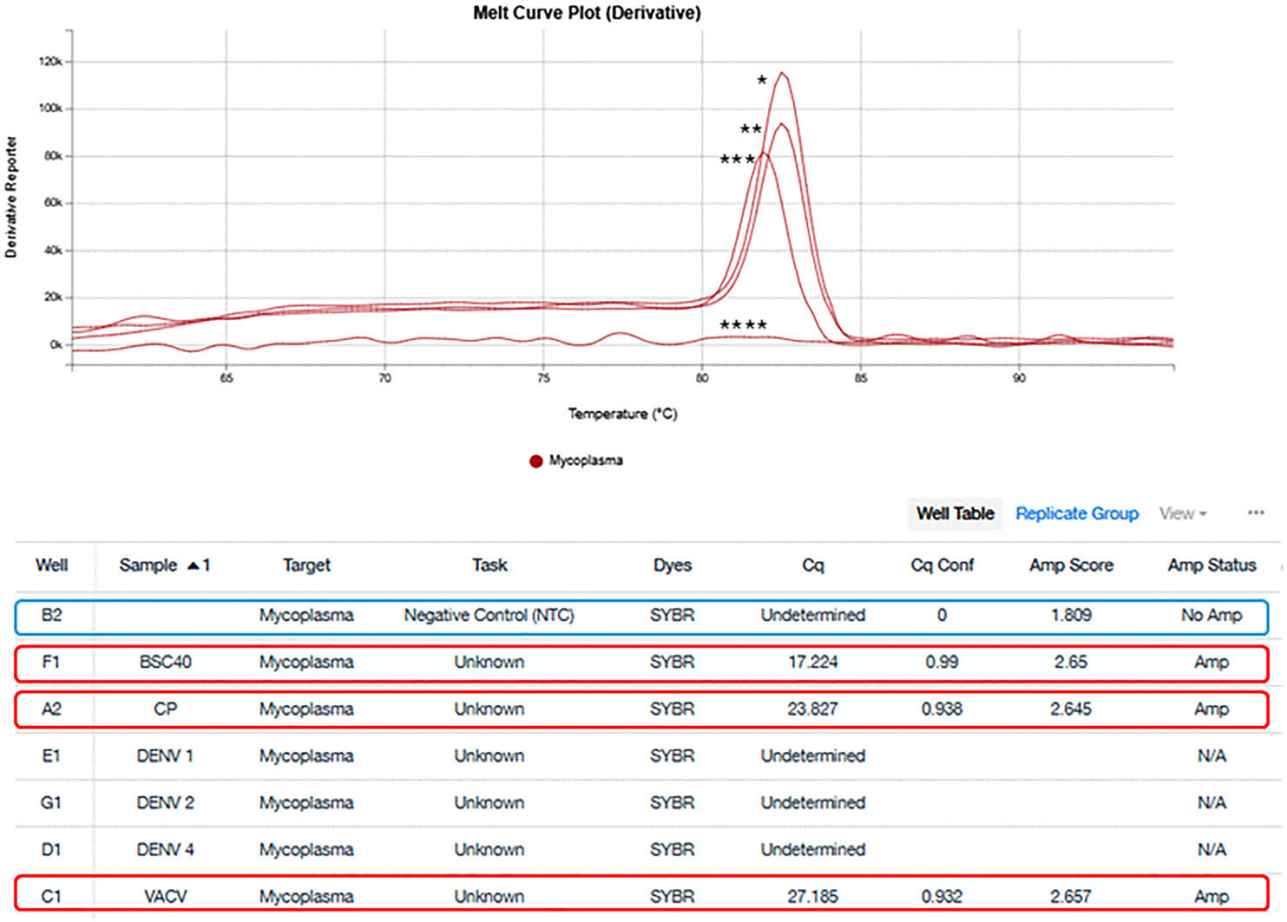
Representative qPCR melting‐curve analysis for Mycoplasma detection. The melting‐curve plot shows specific amplification in the BSC‐40 cell sample (*), the Vaccinia virus stock (**), and the positive control (***), with characteristic melting peaks at approximately 82–83°C. The no‐template control (NTC; ****) showed no amplification. The DENV‐1, DENV‐2, and DENV‐4 samples, included as additional viral samples, also showed no Mycoplasma amplification. The accompanying well table presents the Cq values and amplification status for each sample. CP, positive control; NTC, no‐template control; Cq, quantification cycle.

#### Expected results for Basic Protocol 2 (Treatment of contaminated cultures)

Following antibiotic treatment, a progressive reduction in *Mycoplasma* load is expected across successive cell passages. In intermediate qPCR analyses, this reduction may manifest as increased *Ct* values or decreased amplification signal intensity.

At the end of the full treatment cycle, successfully decontaminated cultures should show no specific qPCR amplification, while positive and negative controls remain valid. Sustained absence of amplification after multiple passages is the primary criterion for confirming treatment success.

Throughout the treatment period, cell morphology and growth must be closely monitored (Figure 2). Successfully recovered cultures maintain morphology consistent with expected standards, exhibit stable growth rates, and show no severe cellular stress. Persistent morphological alterations, markedly reduced viability, or slow growth may indicate treatment‐associated toxicity or irreversible damage to the culture.

**Figure 2:**
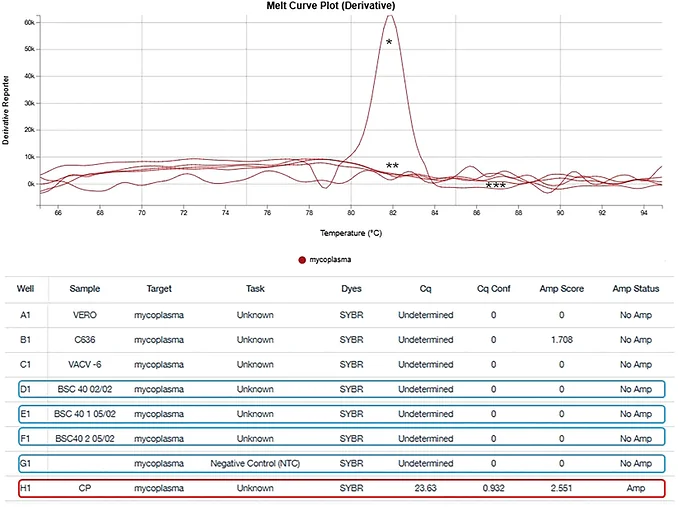
qPCR results from BSC‐40 cell aliquots. This figure presents data from the same qPCR run shown in Figure 3, with emphasis on the BSC‐40 cell samples. Specific amplification was observed only for the positive control (CP; *), with a Cq value of 23.63. No amplification was detected in the three BSC‐40 cell aliquots collected on different dates (**), the no‐template control (NTC; ***), or the VERO, C636, and VACV‐WR 10⁻⁶ samples included in the same run. The accompanying well table presents the Cq values and amplification status for all samples. CP, positive control; NTC, no‐template control; Cq, quantification cycle.

#### Expected results for Basic Protocol 3 (Complementary procedures and residual Mycoplasma reduction)

Additional procedures, such as serial dilutions of biological material or subsequent passages under controlled conditions, further reduce residual *Mycoplasma* burden. The underlying rationale is that dilution of contaminated material leads to a concomitant decrease in the associated microorganism.

Successful outcomes are characterized by continued negative qPCR results across multiple passages or dilutions, with no reappearance of specific amplification (Figure 3). The stability of these results confirms that the absence of *Mycoplasma* is not transient and that decontamination has been effective.

**Figure 3:**
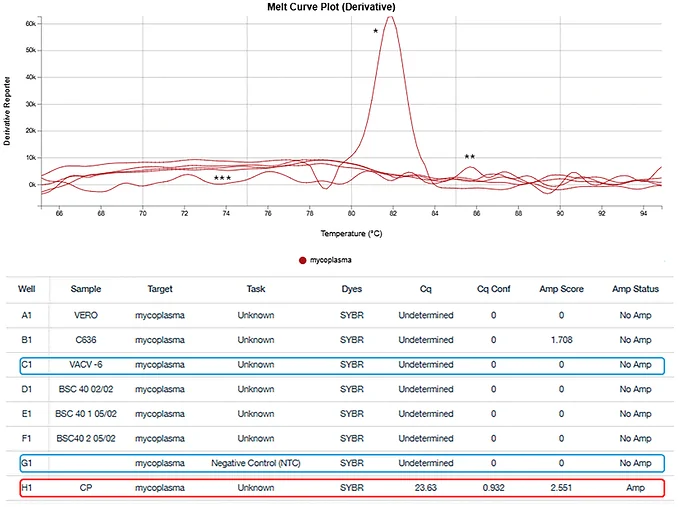
qPCR result following treatment of the VACV‐WR stock. This figure presents data from the same qPCR run shown in Figure 2, with emphasis on the VACV‐WR 10⁻⁶ dilution. Specific amplification was observed only for the positive control (CP; *), with a Cq value of 23.63. No amplification was detected in the VACV‐WR 10⁻⁶ sample (**), the no‐template control (NTC; ***), or the VERO, C636, and BSC‐40 samples included in the same run. The VACV‐WR 10⁻⁶ dilution was the highest dilution that showed cytopathic effect during cell‐culture monitoring and tested negative for Mycoplasma; the cytopathic effect is not shown in this figure. This dilution was therefore selected for viral expansion and production of a new viral stock. The accompanying well table presents the Cq values and amplification status for all samples. CP, positive control; NTC, no‐template control; Cq, quantification cycle.

#### Negative results and unexpected findings

In some cases, specific amplification may persist despite completion of treatment cycles, indicating *Mycoplasma* resistance, cross‐contamination, or biosafety failures. Under these circumstances, discontinuing culture use and safely disposing of the material are recommended.

Unexpected findings include late reappearance of amplification after prolonged periods without detection, often associated with low residual contaminant levels or accidental reintroduction during subsequent handling. These outcomes underscore the importance of periodic monitoring and strict physical separation between contaminated and *Mycoplasma*‐free cultures.

### Time Considerations

The time required to complete the protocols varies depending on the number of samples and the initial condition of the cultures:

• Basic Protocol 1 (qPCR detection): approximately 1 day, including DNA extraction, reaction setup, and qPCR run.
• Basic Protocol 2 (Antibiotic treatment): approximately 3–4 weeks, considering six consecutive cell passages.
• Basic Protocol 3 (Complementary procedures): variable, typically ranging from 3 to 7 additional days, depending on the number of passages or dilutions performed.

The total time from initial detection to confirmation of *Mycoplasma*‐free status generally ranges from 4 to 5 weeks.

### Author Contributions

**Eliza Flores‐Souza**: Conceptualization; data curation; formal analysis; investigation; methodology; writing—original draft; writing—review and editing. **Carolina Colombelli Pacca‐Mazaro**: Conceptualization; data curation; formal analysis; investigation; methodology; writing—original draft; writing—review and editing. **Naiara Clemente Tavares**: Conceptualization; data curation; formal analysis; investigation; methodology. **Camila Salles Nascimento**: Conceptualization; formal analysis; investigation; methodology; writing—review and editing. **Tamiris Vanessa Miguel de Souza**: Conceptualization; investigation; methodology. **Anna Beatriz de Oliveira Mendes**: Data curation; funding acquisition; investigation; writing—review and editing. **Erna Geessien Kroon**: Investigation; methodology. **Carlos Eduardo Calzavara‐Silva**: Data curation; formal analysis; funding acquisition; project administration; writing—review and editing. **Jaquelline Germano de Oliveira**: Conceptualization; data curation; formal analysis; funding acquisition; investigation; project administration; resources; supervision; writing—review and editing.

### Conflict of Interest

The authors declare that they have no financial or personal relationships that could have influenced or biased the work reported in this manuscript. The authors also declare no affiliation with organizations that sell or provide reagents used in the protocols described.

## Data Availability

The data, tools, and materials (or their source) that support the protocol are available from the corresponding author upon reasonable request.
